# Optimization of Ultrasound-Assisted Extraction Process for Silkworm (*Antheraea pernyi*) Pupae Protein and Its Impact on Functional and Structural Characteristics of Protein

**DOI:** 10.3390/molecules30173580

**Published:** 2025-09-01

**Authors:** Yuanyuan Zeng, Hanyu Guo, Yingying Li, Yinghao Xu, Mengli Zhang, Cancan Luo, Yanan Zang, Ji Luo

**Affiliations:** School of Life Sciences, Anhui Normal University, Wuhu 241001, China; 13557506257@163.com (Y.Z.); ghyshitiancai@gmail.com (H.G.); 2421011789@ahnu.edu.cn (Y.L.); xu080512138@163.com (Y.X.); zhangmengli@ahnu.edu.cn (M.Z.); luocancan00@ahnu.edu.cn (C.L.); zang.yn@ahnu.edu.cn (Y.Z.)

**Keywords:** silkworm (*Antheraea pernyi*) pupae protein, ultrasound, optimization, functional characteristics, structure

## Abstract

In this study, the ultrasonic-assisted extraction of silkworm pupae protein (SPP) was optimized using response surface methodology. Subsequently, the effects of ultrasonic treatment on the structural and functional characteristics of SPP were systematically analyzed and verified through Pearson correlation analysis. The results showed that the optimal extraction parameters were an ultrasonic treatment time of 120 min, a power of 115 W, a temperature of 54 °C, pH of 10.5, and the average extraction yield was 68.087%. Compared to the control, ultrasonic treatment significantly improved the functional properties of SPP, including solubility (13.13 g/L), water holding capacity (0.18%), oil holding capacity (0.28%), foaming capacity (55.35%), foam stability (12.71%), emulsification activity (2.15 m^2^/g), emulsification stability (21.95%), gel water holding capacity (11.5%), gel hardness (1.02 N), and gel elasticity (0.49 mm). In addition, the adsorption ability of SPP for 2-octanone and aldehyde was enhanced after ultrasonic treatment. Furthermore, the absorption intensity and maximum wavelength of the SPP fluorescence spectrum extracted via ultrasonic treatment were enhanced, along with the increased surface hydrophobicity and more stable secondary structure which contributed to promoting the functional properties of SPP, proven by Pearson correlation analysis. This study provides a theoretical basis for the further utilization of SPP in the food industry.

## 1. Introduction

Silkworm pupae, the pupal stage of sericigenous insects (*Bombyx mori* and *Antheraea pernyi*), are a valuable by-product of the silk industry. China, as the origin of tussah silkworm pupae (*Antheraea pernyi*), produces over 100,000 metric tons annually, accounting for >90% of global output [1]. Life-cycle assessments indicated that pupae cultivation used 75–85% less land and emitted 40–60% fewer greenhouse gases than conventional livestock systems [2]. These properties make silkworm pupae a sustainable, nutrient-rich protein source suitable for human food [3]. Torres et al. [4] incorporated silkworm pupae powder (SPP) into biscuit formulations and demonstrated that a 15% SPP substitution significantly increased protein content by 12.55% compared to conventional biscuits. David et al. [5] found that the addition of 4% SPP could increase the hardness and adhesion of ice cream, enhanced the functional characteristics of ice cream and kept the proper taste of ice cream. Akande et al. [6] used SPP to prepare functional protein biscuits, which could promote antibacterial activity and flavor quality. Kim et al. [7] replaced 10% of the lean meat in the emulsified sausage with SPP to serve as a new protein component, proving that SPP could improve the cold resistance and yield of sausages.

In recent years, according to the characteristics of “cavitation phenomenon”, “mechanical vibration”, and “thermal effect” [8], ultrasonic-assisted extraction technology has been widely used in the food processing industry. The cavitation effect serves as the primary driving force during extraction [9]. Throughout the ultrasonic process, cavitation bubbles undergo oscillation, growth, contraction, and repeated collapse, generating intense turbulence and high-velocity particle collisions, thereby affecting protein structure [10]. Studies demonstrated that protein structural disruption could significantly enhance extraction yield [11]. It was found that ultrasound treatment not only increased the extraction yield of peanut protein, but also improved the emulsifying and foaming properties of peanut protein [12]. Research has demonstrated that ultrasonic treatment could induce structural changes in SPP, thereby enhancing its functional properties [13]. Phuangjit et al. [14] compared ultrasound, microwave, and freeze-thaw-assisted extraction of SPP and found that ultrasound extraction improved protein emulsification, foaming activity, protein extraction efficiency, and antioxidant activity. To improve the functional and structural properties of the protein and promote the processing performance of SPP, this study employed ultrasound-assisted extraction of SPP based on alkaline dissolution and acid precipitation.

Proteins could adsorb flavor substances, addressing challenges in their dispersion within hydrophilic systems [15]. Yu et al. [16] demonstrated that ultrasound treatment could improve the structural and functional properties of myofibrillar protein (MP) and enhance its binding capacity to furan compounds. Additionally, ultrasound treatment facilitated the binding of myosin to heptanal/hexanal, thereby enabling myosin to retain volatile flavor compounds [17]. However, current research on insect proteins as natural carriers for adsorbing flavor compounds remains limited. Therefore, this study also investigated the adsorption capacity of SPP to the flavor compounds.

In this experiment, RSM was utilized to optimize the ultrasound-assisted extraction process of SPP. The impact of ultrasonic treatment on the functional properties of SPP was investigated by evaluating its solubility, oil-holding capacity, foaming ability and foam stability, emulsion stability, and flavor compound adsorption characteristics. Meanwhile, the relationship between the structural alterations in SPP induced by ultrasonic treatment and its pivotal functional characteristics have been systematically investigated. Furthermore, we also integrated the adsorption capacity of flavor substances into the research framework of ultrasonic-modified proteins, offering a crucial foundation for utilizing ultrasonic technology to produce SPP with more tailored flavors.

## 2. Results and Discussion

### 2.1. Single-Factor Experiment

**Figure 1:**
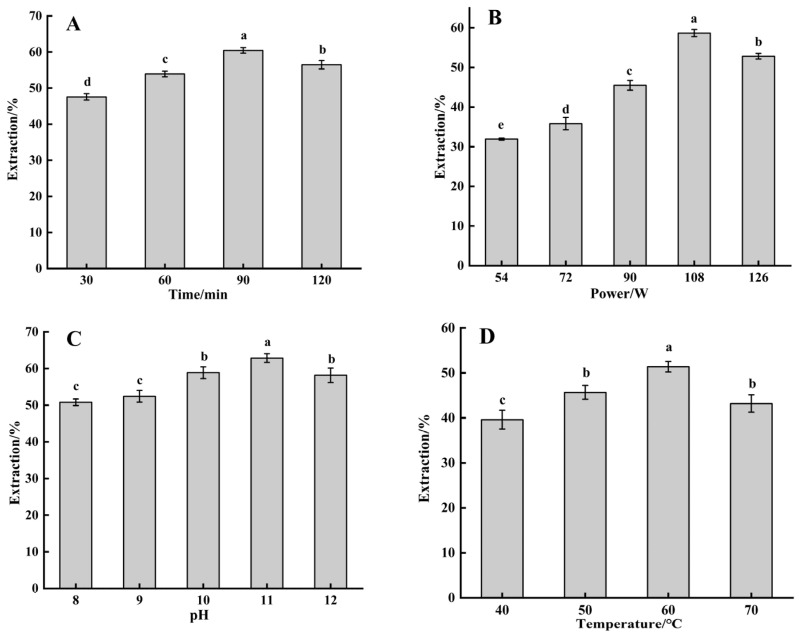
Effects of different extraction parameters on SPP extraction yield. (**A**) The effect of ultrasound time on SPP extraction. (**B**) The effect of ultrasound power on SPP extraction. (**C**) The effect of pH on SPP extraction. (**D**) The effect of ultrasound temperature on SPP extraction. Data were expressed as means ± SEM. Labeled characters with different letters represent significant differences at *p* < 0.05.

The effects of ultrasonic time, power, temperature, and solution pH on protein extraction efficiency were investigated through single-factor experiments. As shown in Figure 1, the extraction yield initially increased before subsequently decreasing under all experimental conditions. As shown in Figure 1A, the extraction yield initially increased with prolonged ultrasonication time, which was attributed to the disintegration effects of acoustic cavitation. The ultrasonic wave fragmented protein molecules into smaller aggregates, thereby improving extraction efficiency [18]. However, extended treatment time beyond the optimal duration reduced extraction yields, possibly due to excessive shear forces from cavitation-induced protein degradation and oxidative damage, leading to insoluble aggregate formation [19]. As shown in Figure 1B, the observed increase in extraction yield might have resulted from enhanced cavitation effects at higher ultrasonic power levels. This mechanical action could modify protein conformation [20], disrupt insoluble protein aggregates and promote the reorganization of proteins into soluble aggregates, which improved extractability [21]. The subsequent decrease in protein extraction efficiency at higher power levels might be attributed to protein structural degradation induced by intensified thermal and mechanical effects associated with high-intensity ultrasonication [22]. As shown in Figure 1C, the increase in pH induced the unfolding of protein molecules, exposing more hydrophobic regions [23]. Ultrasonic cavitation further disrupted the higher-order structure of proteins, inhibiting their refolding [24], while simultaneously enhancing protein solubility and extraction efficiency. Under highly alkaline conditions, protein denaturation occurred and disrupted secondary and tertiary structures. The cleavage of disulfide bonds within proteins could lead to structural unfolding or loosening [25], which promoted the formation of insoluble aggregates and subsequently reduced extraction efficiency. As shown in Figure 1D, the enhanced extraction yield could be attributed to the elevated temperature, which increased cavitation bubble nucleation. This temperature-dependent effect promoted bubble population density, accelerated mass transfer rates and improved extraction yield [26]. However, overexposure of proteins to high temperatures might result in the degradation of their molecular structures [27]. Furthermore, elevated temperature could suppressed cavitation activity by reducing liquid surface tension and vapor pressure [26], which ultimately diminished extraction efficiency.

Based on the single-factor experimental results, the following ranges were selected for further optimization: ultrasonic time (60, 90, and 120 min), power (90, 108, and 126 W), solution pH (10, 11, and 12), and temperature (50, 60, and 70 °C).

### 2.2. RSM Data Analysis

The RSM design and corresponding results for SPP extraction yield are shown in Table 1. A quadratic polynomial model described the relationship between the extraction yield (Y) and the independent variables, including ultrasonic time (A), power (B), pH (C), and temperature (D). The fitted model was expressed as follows: Y = 67.65 + 1.19A + 0.9908B − 1.4C + 1.37D − 0.3725AB − 0.1200AC − 2.02AD + 0.7100BC − 3.65BD + 1.77CD − 1.42A^2^ − 2.89B^2^ − 2.21C^2^ − 2.60D^2^.

### 2.3. Analysis of Variance of the RSM Regression Model

As shown in Table 2, the model illustrated high statistical significance (*p* < 0.0001), indicating that the SPP extraction yields were significantly different among the experimental conditions. The misfit term of the model was 0.4555 (*p* > 0.05), indicating that the model had a good fit. This demonstrated that the model was meaningful and the comprehensive model was accurate and reliable [28]. The correlation coefficient (R^2^ = 0.9291) and the adjusted determination coefficient (R^2^adj = 0.8582) indicated that the model explained 92.91% of the variability of the three factors and accounted for 85.82% of the SPP extraction conditions. Based on the F-value of each single factor, the larger the F-value, the greater the influence of the single factor on SPP extraction [29]. The influence degree of single factors followed the order: C > D > A > B. First-order terms A, C, and D showed extreme significance, while B demonstrated significance. The interaction terms AD, BD, and CD exhibited extreme significance. All quadratic terms showed extreme significance (*p* < 0.01).

### 2.4. Analysis and Optimization of RSM

**Figure 2:**
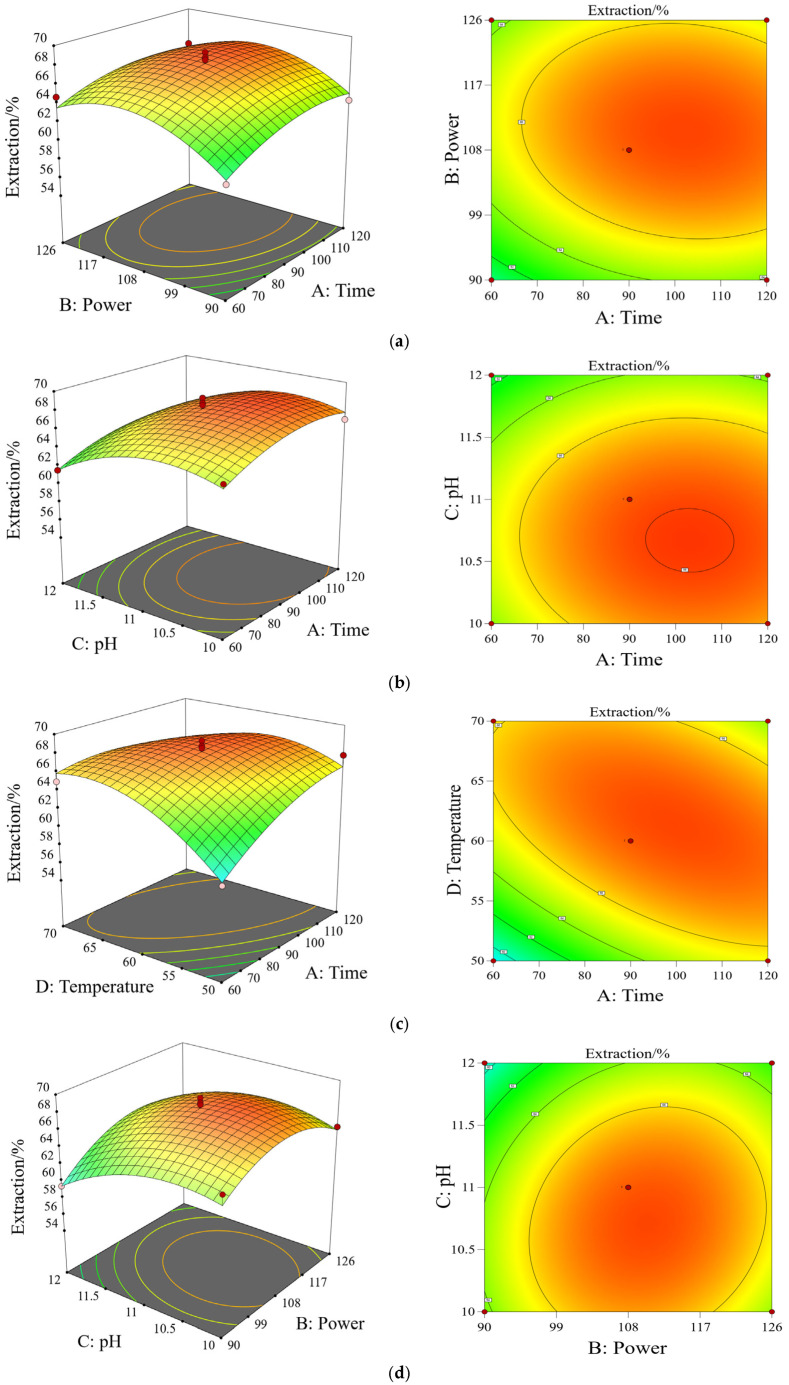

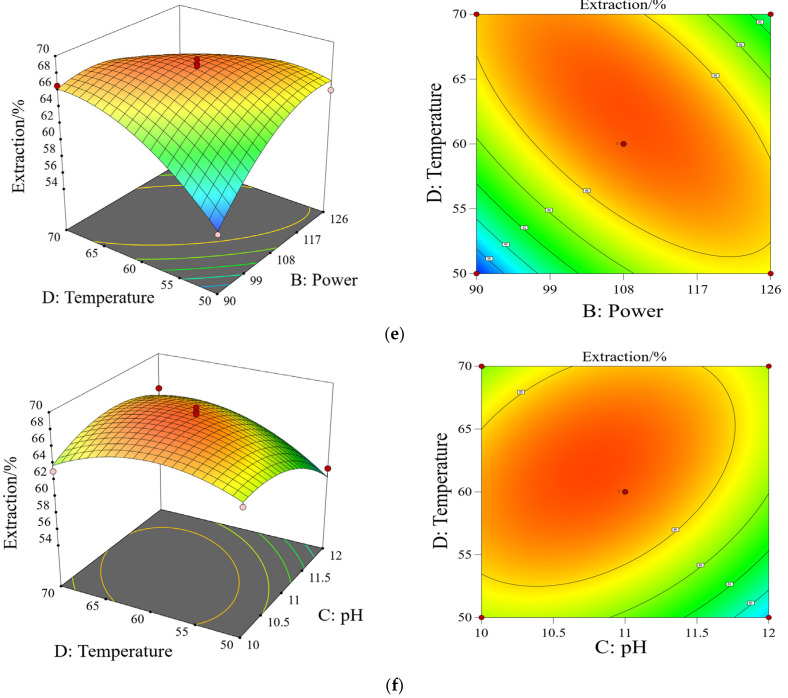
The curve and contour plot of interaction of various factors on the extraction yield of SPP. Note: Response surface and contour plots of the effects of (**a**) ultrasound time and ultrasound power; (**b**) ultrasonic time and pH; (**c**) ultrasound time and temperature; (**d**) Ultrasonic power and pH; (**e**) Ultrasonic power and temperature; (**f**) Ultrasonic temperature and pH.

As shown in Figure 2, the contour plots and RSM analysis revealed significant interactions between the factors. The degree of interaction was indicated by the ellipticity of the contour lines, with more elliptical contours representing stronger interactions. Similarly, the steepness of the RSM reflected the intensity of the factor effects, where steeper surfaces correspond to more pronounced interactions [30]. The RSM results showed that with increasing levels of A, B, C, and D, the SPP extraction yield first increased and then decreased, which was consistent with the results presented in Table 2. The optimal SPP extraction conditions obtained through RSM screening were as follows: an ultrasonic time of 120 min, a power of 115 W, a temperature of 54 °C, and pH of 10.5. The predicted theoretical extraction yield of SPP under optimized conditions was 68.139%. To verify the optimization, three parallel experiments were conducted under an ultrasonic time of 120 min, a power of 115 W, a temperature of 54 °C, and pH of 10.5. The average SPP extraction yield from three parallel experiments reached 68.087%, which closely matched the predicted value (68.139%). This confirmed the adequacy of the response model for the optimization process [31]. And compared with the untreatment group (60.977%), the extraction yield significantly increased by 7.11% (*p* < 0.05).

### 2.5. Functional Characteristic Analysis

#### 2.5.1. Solubility

The effects of ultrasonic treatment on the functional characteristics of SPP are summarized in Table 3. Compared with the untreated SPP, the ultrasonically treated SPP showed a 1.3-fold increase in solubility (*p* < 0.05). The increase in solubility might result from the disruption of non-covalent interactions (e.g., hydrogen bonds and hydrophobic interactions) induced by the oxidation and mechanical shear effects caused by ultrasonic treatment, leading to the release of smaller, more soluble protein fragments [32]. These fragments enhanced water−protein interactions, resulting in improved solubility.

#### 2.5.2. WHC, OHC, EAI, and ESI

The WHC, OHC, and gel water retention of SPP were significantly enhanced after ultrasonic treatment. This improvement could be attributed to the disruption of intramolecular interactions (e.g., disulfide bonds and van der Waals forces) induced by ultrasound [33], which led to the expansion of protein conformation and increased surface accessibility [34]. The cavitation effect enhanced protein adsorption at the air−water interface, improving the foaming performance and foam stability, which were associated with increased surface hydrophobicity and reduced protein particle size [35].

The EAI and ESI enhancements likely resulted from ultrasonication-induced structural modifications: the 5.6% increase in α-helix content (Table 4) enhanced protein rigidity [36], in the subsequent experiment of Section 2.6, it was also demonstrated that thenewly exposed hydrophobic groups improved interfacial adsorption [37].

#### 2.5.3. Adsorption Capacity of Flavor Compounds

**Figure 3:**
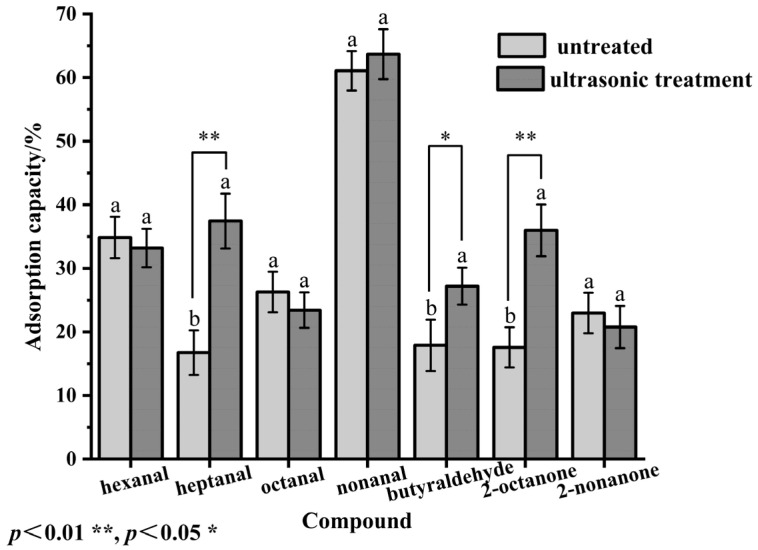
Effect of ultrasonic treatment on the adsorption capacity of flavor compounds. Data were expressed as means ± SEM. Labeled characters with different letters represent significant differences at *p* < 0.05.

The adsorption capacities of the SPP solution before and after ultrasonic treatment to hexanal, heptanal, octanal, nonanal, butyraldehyde, 2-octanone, and 2-nonanone were studied. These aldehydes and ketones are key flavor compounds or odor-active components commonly found in food systems [38]. Heptanal, nonanal, and hexanal are typical volatile flavor compounds of silkworm (*Antheraea pernyi*) pupae [39]. As shown in Figure 3, the adsorption capacity of treated SPP for heptanal, nonanal, and butyraldehyde increased, while the adsorbability for hexanal and octanal decreased slightly. The binding ability of SPP to five aldehydes did not increase with carbon chain length, which might be caused by ultrasonic cavitation. This effect constantly dispersed or aggregated the SPP structure, causing its binding sites to be alternately exposed or hidden [32]. The adsorption capacity of 2-octanone was enhanced, owing to the factors such as carbon atom count, chain length, and branching degree [40]. Heptanal and nonanal are long-chain aldehydes with high hydrophobicity. After ultrasonic treatment, the surface hydrophobicity of the protein increased due to exposed hydrophobic regions that were primarily composed of nonpolar amino acid residues (e.g., leucine, phenylalanine) [41]. These hydrophobic regions preferentially interacted with non-polar molecules [42], such as heptanal and nonanal. The resulting hydrophobic interactions caused better adsorption capacity. In contrast, hexanal and octanal exhibited higher polarity, particularly octanal, which contained a carboxyl group. This polar functional group resulted in low affinity with the hydrophobic protein surface [43], which slightly decreased their adsorption capacity after ultrasonic treatment.

### 2.6. Structural Characteristic Analysis

#### 2.6.1. SDS-PAGE Analysis

**Figure 4:**
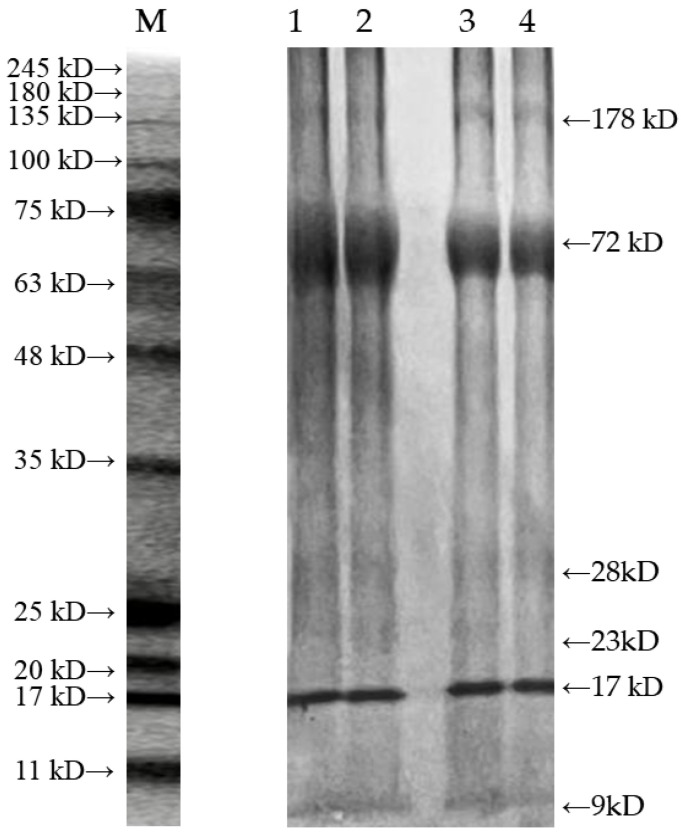
SDS-PAGE profiles of SPP treated with or without ultrasonication. Note: M is the standard high-molecular-weight maker; both 1 and 2 are SPP after ultrasonic treatment; 3 and 4 are both untreated SPP.

As presented in Figure 4, six obvious bands with molecular weights of 9, 17, 23, 28, 72, and 178 kDa were obtained, respectively. Among them, the 28 kDa band was the livetin of *Bombyx mori*, the 72 kDa band was a subunit of egg-specific protein of silkworm (*Antheraea pernyi*) pupae, the 178 kDa band was a large subunit of vitellogenin and vitellophosphoprotein [44], and the 9 kDa fragment likely originated from ultrasound-induced cleavage of peptide bonds, generating low-molecular-weight peptides. Compared with the untreated SPP, the electrophoretic bands of SPP after ultrasonic treatment did not change significantly. This demonstrated that peptide bonds remained intact during ultrasonic treatment and shear exposure, which was consistent with the previous results, such as the primary structure of ovalbumin [45], walnut protein [32], and soy protein isolate [19]. However, the peptide spectrum appeared blurred, which might be caused by ultrasonic turbulence and cavitation effects [46]. These mechanical forces likely induced slight cleavage of high-molecular-weight peptides.

#### 2.6.2. Changes in Secondary Structure of SPP

**Figure 5:**
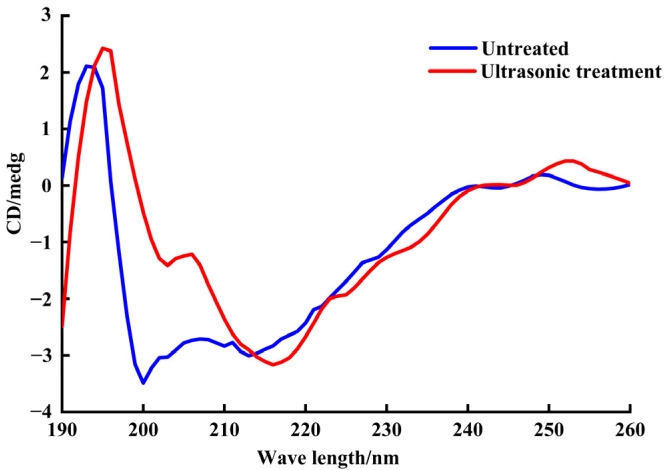
Effect of ultrasonic treatment on the secondary structure of SPP.

The circular dichroism spectra of SPP extracted by different treatments are shown in Figure 5. The SPP extracted by ultrasound showed an obvious positive peak in the range of 185–200 nm and a negative peak at 218 nm, which were characteristic peaks of the β-sheet structure. It could be seen from Table 4 that after the ultrasound treatment, the content of α-helix increased by 5.6%, β-turn increased by 8.6%, and the content of β-sheet and random coil decreased by 13.6% and 0.7%, respectively, which was consistent with the results of Hu et al. [19]. The observed differences in secondary structure content resulted from shear forces and cavitation effects generated during ultrasonication, which could disrupt intermolecular interactions and induce conformational rearrangements within protein molecules [19]. The β-sheet structures were converted into stabilized α-helices. Since α-helices are stabilized by intramolecular hydrogen bonds, whereas β-sheets rely on intermolecular hydrogen bonds, ultrasonic treatment promoted the reconstruction of intramolecular hydrogen bonds in SPP [47]. The increase in α-helix content and decrease in β-sheet content could lead to contraction of the SPP peptide chain, consequently enhancing hydrophobicity [48]; this structural change might expose interior hydrophobic residues.

#### 2.6.3. Surface Hydrophobicity Analysis

**Figure 6:**
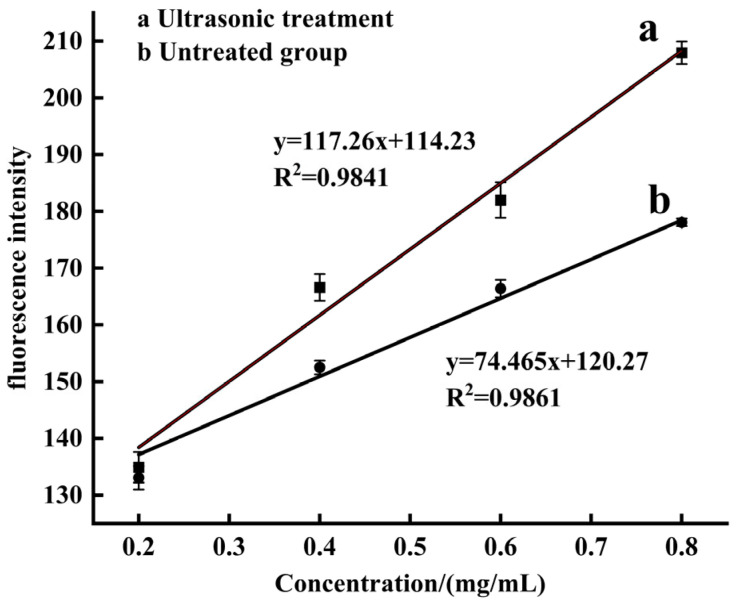
Effect of ultrasonic treatment on surface hydrophobicity of SPP.

Protein surface hydrophobicity is a critical indicator of the number of hydrophobic groups exposed to a polar environment [41]. In Figure 6, ultrasonic treatment elevated the S_0_ value by 57.5%, from 74.465 ± 2.64 to 117.26 ± 1.35 (*p* < 0.05), indicating the surface hydrophobicity of SPP was significantly increased by 1.6-fold after ultrasonic treatment, which could be caused by acoustic cavitation, generated localized high temperatures, high pressures, and intense shear forces, disrupting the higher-order structure of proteins [49], inducing the unfolding of the protein, and exposing hydrophobic groups [50]. This structural modification not only enhanced the surface hydrophobicity but also improved the emulsifying capacity of SPP (Table 3) [51], as hydrophobic regions played a key role in adsorbing at the oil−water interface and stabilizing emulsion droplets [52]. In this study, we observed a positive correlation between surface hydrophobicity and SPP solubility, which was consistent with the finding of Li et al. [53]. This phenomenon was governed by the equilibrium between attractive and repulsive forces among protein molecules, which depended on ultrasound-induced intermolecular conformational changes. Within the optimal ultrasonic power range, the molecular structure of insoluble protein aggregates became looser, promoting the reorganization of soluble proteins. Meanwhile, ultrasonication simultaneously reduced particle size, expanded interfacial contact area and enhanced protein−water interactions, collectively improving protein solubility. However, when ultrasonic intensity exceeded the optimal range, excessive protein denaturation and insoluble aggregate formation decreased solubility. The experimental results demonstrated that the optimized process conditions in this study effectively enhanced the functional properties of SPP.

#### 2.6.4. SPP Gel Properties Analysis

As shown in Table 5, ultrasonic treatment significantly promoted the gel strength and the elasticity of SPP (*p* < 0.05). This enhancement was attributed to the increased surface hydrophobicity of the protein, which exposed more hydrophobic groups and promoted intermolecular and intramolecular interactions, resulting in facilitated formation of protein aggregates [54]. These aggregates contributed to the development of a stronger and more elastic gel network. In addition to gel strength, the WHC of gel was also significantly increased after ultrasonic treatment (*p* < 0.05) due to the more compact and orderly arrangement of molecules within the gel network, which formed finer pores and channels [55]. These structural changes increased the contact area between the gel matrix and water molecules, as supported by previous study [56], and indicated a synergistic effect of ultrasonic treatment on the functional properties of SPP.

#### 2.6.5. Scanning Electron Microscopy of SPP

**Figure 7:**
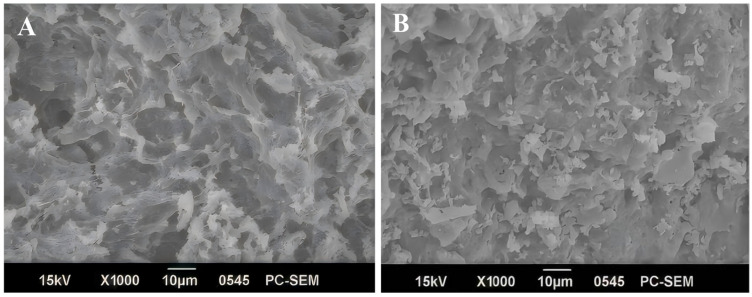
Scanning electron microscopy micrographs of the untreated (**A**) and ultrasonically treated (**B**) SPP. Scale bars indicate 10 μm.

To further verify the above conclusions, the microstructure of SPP gel was observed by scanning electron microscope (SEM). The SPP surface pores of ultrasonic treatment exhibited smaller dimensions compared to those of untreated SPP (Figure 7). The rearrangement of protein molecules induced by ultrasonic cavitation and mechanical vibration could reduce irregular structure and enhance the mechanical strength of the gel. This process resulted in smaller and more uniformly distributed pores, showing that ultrasonic treatment improved intermolecular cross-linking, resulting in a more dense network structure [57]. This structure could physically entrap water molecules, enhancing the WHC of the gel [58], which was consistent with the previous results in this study.

### 2.7. Correlation Analysis Between the Functional and Structure of SPP After Ultrasonic Treatment

**Figure 8:**
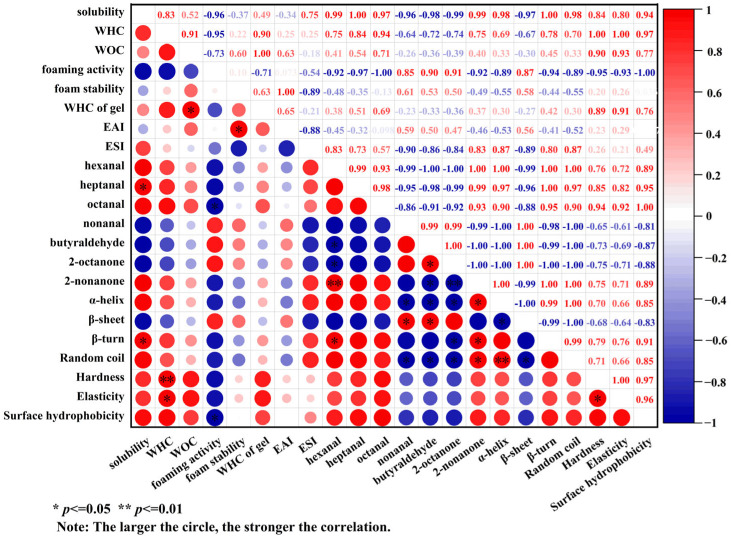
Correlation between the function and structure of SPP after ultrasonic treatment.

Figure 8 presents the correlation results between ultrasonic treatment, protein structure, and the functional properties of the protein. The WHC of the protein was closely associated with its gel properties, showing a significant positive correlation not only with gel strength (*p* < 0.01) but also with gel elasticity (*p* < 0.05). This phenomenon could be attributed to the regulatory effect of ultrasound treatment on protein structure. Ultrasound treatment promoted the expansion of protein molecules, exposed more hydrophilic groups and enhanced intermolecular crosslinking, thereby facilitating the formation of a more uniform three-dimensional network structure. The uniform gel network effectively immobilized water molecules, reduced water loss and significantly enhanced the WHC, mechanical strength, and elasticity of the gel. Furthermore, ultrasonic treatment induced the changes in protein secondary structure through the synergistic effect of mechanical forces, thermal effects, and free radical effects generated by cavitation, and remarkably affected the adsorption capacity of flavor substances. Specifically, increased β-turn content showed a positive correlation with protein adsorption capacity for hexanal and 2-nonanone (*p* < 0.05), while increased α-helix content exhibited both positive correlation with 2-nonanone adsorption (*p* < 0.05) and negative correlations with nonanal, butyraldehyde, and 2-octanone adsorption (*p* < 0.05). These results demonstrated that ultrasonic treatment significantly altered the flavor adsorption capacity of SPP. Moreover, ultrasound improved protein solubility, which was significantly positively correlated with the increase of β-turn (*p* < 0.05). Because protein solubility directly affected the adsorption efficiency between protein and flavor substances, this structure−function relationship further explained the regulatory mechanism of ultrasound treatment on the flavor adsorption performance.

## 3. Materials and Methods

### 3.1. Materials and Reagents

Silkworm (*Antheraea pernyi*) pupae were purchased from Qingdao City, Shandong Province, in October 2024. The pupae were bisected longitudinally, freeze-dried for 48 h and pulverized using a grinder. The resulting silkworm pupae powder was sieved through a 60-mesh screen and stored at −18 °C until use.

Petroleum ether, 2.5% glutaraldehyde solution, tert-butyl alcohol, ammonium 8-anilino-1-naphthalene sulfonate (ANS), and sodium dodecyl sulfate (SDS) (analytical grade) were obtained from Shanghai Sinopharm Chemical Reagent Co., Ltd. (Shanghai, China); Bovine Serum Albumin (BSA), Mas Brilliant Blue G-250, and Tris-Glycine Electrophoresis Buffer were obtained from Shanghai Yuanye Biotechnology Co., Ltd. (Shanghai, China); Rainbow 245 broad-spectrum protein marker and 4 × protein loading buffer (with sulfhydryl reducing agent) were obtained from Solarbio Science & Technology Co., Ltd. (Beijing, China); FuturePAGETM precast protein gels were obtained from Nanjing Aisiyi Biotechnology Co., Ltd. (Nanjing, China); chromatography-grade volatile compounds: octanal, butyraldehyde, hexanal, heptanal, nonanal, 2-nonanone, and 2-octanone were obtained from Shanghai Sinopharm Chemical Reagent Co., Ltd. (Shanghai, China). 

### 3.2. Defatted Silkworm Pupae Powder

Due to the influence of fat content on the functional structural characteristics of silkworm pupa protein, a defatting process was carried out [59]. Referring to the method of Mishyna et al. [60], the silkworm pupae powder (10 ± 0.02 g) was weighed and mixed with petroleum ether as the ratio of 1:10 (*w*/*v*), oscillated at 160 r/min for 120 min and then centrifuged for 10 min at 12,000 r/min (TGL-20M, Centrifuge, Hunan Xiangyi Laboratory Instrument Development Co., Ltd., Changsha, China). After that, the supernatant was taken and recovered by rotary evaporation (RE-5299 Rotary evaporator ShangHai Ya Rong instrument Co., Ltd., Shanghai, China). The obtained silkworm pupae powder was placed in a ventilated place for 12 h to obtain defatted silkworm pupae powder.

### 3.3. Ultrasonic-Assisted Extraction of SPP

SPP was extracted by alkali dissolution and acid precipitation according to Hu et al. [61]. The silkworm pupae powder was dissolved in water according to the material/liquid ratio of 1:12 (*w*/*v*), pH was adjusted to 10 using 1 M NaOH, and then, the solution was sonicated using an ultrasonic water bath instrument (SN-QX-65 Shanghai Shangyi Technology Co., Ltd., Shanghai, China), with the ultrasonic frequency fixed at 40 kHz. The sonicated solution was filtered through gauze. The filtrate was collected, and the pH was adjusted to 4.5 with 1 M HCl. After stirring for 5 min, it was centrifuged at 10,000 r/min for 10 min to collect the sediment. The sediment was freeze-dried (FD-2 Vacuum freeze dryer, Shanghai Bilang Instrument Manufacturing Co., Ltd., Shanghai, China) to obtain SPP powder, which was stored in −18 °C for future use.

### 3.4. Determination of Protein Concentration

The standard curve was drawn with bovine serum protein content (μg) as the abscissa and absorbance as the ordinate. The standard curve fitting equation was shown as follows: y = 2.615x + 0.0295; its linear correlation coefficient was 0.9969. Then, 0.5 mL SPP alkali solution and 1.5 mL distilled water were mixed. After that, 3.0 mL Coomassie Brilliant Blue G-250 solution was added and allowed to stand for 2 min. The OD value was measured at 595 nm (UV5100 Ultraviolet-Visible Spectrophotometer, Shanghai Yuan Analysis Instrument Co., Ltd., Shanghai, China), and the protein concentration was calculated according to the standard curve. SPP extraction yield was calculated according to Formula (1) [62]:

(1) $$SPP\;extraction\;\mathrm{yield}/\%=\frac{m_{1}\times {w\prime }_{1}}{m_{2}\times {w\prime }_{2}}\times 100$$

where m_1_ and w′_1_ are the weight of SPP powder (g) and protein content (g/g), respectively; m_2_ and w′_2_ are the weight of defatted silkworm pupae powder (g) and protein content (g/g), respectively.

### 3.5. Single-Factor Experiment and RSM Optimization of Ultrasonic Extraction

#### 3.5.1. Single-Factor Test

The ultrasonic-assisted extraction process was optimized with protein extraction yield as an evaluation index. Experimental parameters were adapted from Ni et al. [39] with minor modifications according to laboratory equipment availability. Preliminary results indicated that lower ultrasonic power could effectively increase this property [63], so we selected a power range of 50–150 W. The exact parameters were determined through systematic optimization experiments and validated through preliminary trials. Ultrasonic power, temperature, time, and reaction pH were selected for the single-factor experiment. The 4 factors were designed as follows: the pH values of SPP solution were 8, 9, 10, 11, and 12; the ultrasonic times were 30, 60, 90, and 120 min; the ultrasonic temperatures were 40, 50, 60, and 70 °C; the ultrasonic powers were 54, 72, 90, 108, and 126 W. The ultrasound time for the first single-factor experiment was set to 60 min, and the other parameters were set to 60 °C, pH 11, and 108 W, respectively [64,65,66]. The best result was selected from the previous single-factor test for the subsequent single-factor test.

#### 3.5.2. RSM Experiment

According to the results of the single-factor test, ultrasonic time (A), power (B), pH (C), and temperature (D) were selected to design the RSM (Table 6).

### 3.6. Determination of Functional Characteristics

#### 3.6.1. Determination of Solubility

Referring to the method of Ni et al. [39], the SPP powder was prepared into 10% suspension, stirred for 30 min at pH 7.0 and centrifuged for 10 min at 12,000 r/min. The protein content of the supernatant was determined by the Coomassie brilliant blue method, and the total protein content of the sample was detected by dissolving it in a NaOH solution. The solubility was calculated with Formula (2):

(2) $$Solubility/\%=\frac{C_{1}}{C_{2}}\times 100\%$$

where C_1_ is the protein content of the supernatant (g/mL); C_2_ is the total protein content (g/mL).

#### 3.6.2. Determination of Water Holding Capacity and Oil Holding Capacity

Referring to the method of Rawdkuen et al. [67], (0.5 ± 0.02) g of SPP powder was weighed, mixed with 10 mL deionized water or tea seed oil, allowed to stand for 30 min and centrifuged at 12,000 r/min for 10 min. Then, the tea seed oil and deionized water were removed from the upper layer, and the total mass was weighed. Water holding capacity (WHC) and oil holding capacity (OHC) were calculated according to Equations (3) and (4):

(3) $$WHC/\%=\frac{\left(m_{3}-m_{2}\right)}{m_{1}}\times 100$$

(4) $$OHC/\%=\frac{\left(m_{4}-m_{2}\right)}{m_{1}}\times 100$$

where m_1_ is SPP powder mass (g); m_2_ is the total mass of the SPP powder and the centrifuge tube (g); m_3_ and m_4_ are the total masses (g) of the centrifuge tube after removing deionized water and tea seed oil from the upper layer, respectively.

#### 3.6.3. Determination of Foaming Activity and Foam Stability

Referring to the method of Phuangjit et al. [14], 5 mL of 1% SPP solution was stirred for 2 min, the foam volume was recorded immediately, and after standing for 10 min, the foam volume was measured again. The foaming activity and foam stability were calculated as Equations (5) and (6):

(5) $$Foaming\;activity/\%=\frac{V_{1}}{V}\times 100$$

(6) $$Foam\;stability/\%=\frac{V_{2}}{V_{1}}\times 100$$

where V is the initial volume of the SPP solution (mL); V_1_ is the foam volume (mL) after stirring for 2 min; V_2_ is the foam volume (mL) after standing for 10 min.

#### 3.6.4. Determination of Emulsifiability and Emulsifying Stability

According to the methodology described by Loushigam and Shanmugam [68], 15 mL of the SPP solution (1 g/L) was mixed with 5 mL of corn oil in a test tube and homogenized at 24,000 r/min for 2 min (JRJ300-DSH High Speed Homogenizer, Shanghai Yunjin Instrument Equipment Co., Ltd., Shanghai, China). Then, 50 μL emulsion was taken from the bottom and diluted 100 times with 1 g/L SDS. The absorbance was measured at a 500 nm wavelength. After the emulsion was placed for 10 min, the preceding operation was repeated. The emulsifying activity index (EAI) and emulsifying stability index (ESI) were calculated according to Formulas (7) and (8):

(7) $$EAI/(m^{2}/g)=\frac{2\times 2.303\times A_{0}\times N}{C\times \varphi \times 10000\times L}$$

(8) $$ESI/(min)=\frac{A_{0}}{∆A}\times ∆t$$

where A_0_ is the absorbance of the diluted emulsion immediately after homogenization; N is the dilution multiple; C is the protein mass concentration (g/mL); φ is the oil volume fraction of the emulsion (%); L is the cuvette thickness (cm); ΔA is the change in absorbance at 0 and 30 min (A_0_–A_30_); Δt is the time interval (min).

#### 3.6.5. Determination of Water Retention of Gels

According to Ma et al. [69], the gel was prepared by dissolving SPP powder to obtain a 8% (*w*/*v*) protein solution, followed by adjusting the pH to 9.0 using 0.1 M NaOH, as determined by preliminary experiments. After 2 mL protein solution was bathed in 85 °C for 30 min, it was immediately cooled with ice water and placed in a 4 °C refrigerator overnight to prepare the gel. After weighing the mass of each gel, it was placed in a 50 mL centrifuge tube with absorbent paper and centrifuged for 5 min at 10,000 r/min. Then, the water was absorbed onto the gel surface and weighed. The water retention capacity was calculated as Formula (9):

(9) $$Gel\;water\;retention/\%=\frac{m_{2}}{m_{1}}\times 100$$

where m_1_ is the mass of the SPP gel sample before centrifugation (g); m_2_ is the mass of the SPP gel sample after centrifugation (g).

#### 3.6.6. Determination of the Adsorption Capacity of Flavor Compounds

With a slight modification referring to Duppeti et al. [70], 0.3 g SPP powder was dissolved in 10 mL 20 mM phosphate buffer at pH 6. Octanal, butyraldehyde, hexanal, heptanal, nonanal, 2-nonanone, and 2-octanone standards were diluted with methanol to 1 mg/mL. Each flavor compound was added to 5 mL of the protein solution. The final concentration was 100 ppm in a 20 mL headspace bottle. The sealed vial was balanced in the dark at 30 °C for 15 h. Then, an 85 μm (CAR/PDMS) solid-phase microextraction needle was used for extraction at 35 °C for 35 min. Finally, the fiber head adsorbing flavor compounds was kept at 220 °C for 5 min at the injection port without diverting samples. The separation valve was closed 1 min after injection, and then, the extract was separated and identified by GC-MS (Qp2010 gas chromatography-mass spectrometry, Shimadzu Instrument Co., Ltd., Kyoto, Japan). The adsorption capacity for flavor compounds was analyzed by the Formulas (10) and (11):

(10) $$Proportion\;of\;flavor\;compounds/\%=\frac{As}{Ac}\times 100$$

(11) Adsorption capacity/%=100−Proportion of flavor compounds

where A_C_ is the peak area of flavor compounds in buffer solution control treatment; A_S_ is the peak area of flavor compounds in the experimental treatment.

### 3.7. Determination of Structural Characteristics

#### 3.7.1. Determination of Texture Characteristics

Referring to Ma, Zhu, and Wang [69], the texture properties of the gel were measured using a texture analyzer (TA.XTC-16 Gel strength tester, Baosheng Technology Co., Ltd., Shanghai, China). Analysis was performed with a P35 probe; the velocities before, during, and after the measurement were all 2 mm/s; the compression deformation rate of the gel was 50%, and the residence time was 3 s.

#### 3.7.2. Scanning Electron Microscopy (SEM)

The SPP sample was sprayed with gold by a gold spraying instrument, and the microstructure of the sample was observed by SEM (Su8010 scanning electron microscope, Hitachi Instruments Co., Ltd., Chiyoda City, Japan).

#### 3.7.3. Determination of Surface Hydrophobicity

Referring to the practice of Zou et al. [20], the SPP solution was diluted to 0.8, 0.6, 0.4, and 0.2 mg/mL with a phosphoric acid buffer solution. Then, 2 mL of the SPP solution at different concentrations was put into a test tube, and subsequently, 10 μL ANS (8 mM) was added into each tube. After reacting for 15 min in the dark at room temperature, a fluorescence spectrophotometer (RF-5310PC fluorescence spectrophotometer, Shimadzu Instrument Co., Ltd., Japan) was used to scan at an excitation wavelength of 374 nm and an emission wavelength of 485 nm, and the slope between the relative fluorescence intensity and corresponding concentration was analyzed by linear regression, which was the surface hydrophobicity S_0_ of each sample.

#### 3.7.4. Secondary Structure Determination

According to Hegde et al. [71], the conditions for circular dichroism scanning (DM245 circular dichroism spectrometer Olis Instruments Co., Ltd., Athens, GA, USA) were as follows: the mass concentration of protein sample was 1 mg/L, the optical diameter of the sample cell was 0.1 cm, the scanning rate was 1 nm/s, the response time was 1 s, and the buffer solution was used as a blank.

#### 3.7.5. SDS-PAGE

Following the method described by Yang et al. [72], a 5 g/L SPP solution was prepared by dissolving SPP powder in 10 mL of 1% (*w*/*v*) NaOH with continuous stirring at 25 ± 1 °C for 2 h. Then, 20 μL of the protein solution was mixed with 10 μL of 2× SDS loading buffer, shaken for 30 s, heated in boiling water (100 °C) for 5 min, cooled to room temperature and then centrifuged for 5 min at 10,000 r/min. Then, 20 μL supernatant and 20 μL marker were injected into the lane. In the first stage, the voltage was adjusted to 80 V, and then, it was added to 135 V after the bromophenol blue was completely moved to the separation gel. When the bromophenol blue migrated to the bottom, the electrophoresis was finished.

### 3.8. Statistical Analysis

Each test was measured three times, and the data were organized by Excel 2021 (Microsoft Corporation, Redmond, WA, USA). SPSS 22.0 (SPSS Inc., Chicago, IL, USA) software was used to analyze the data by one-way ANOVA, and the Duncan’s method was used to test the significance (*p* < 0.05). *T*-test was used to analyze the difference between the two groups of data (*p* < 0.05); Origin 2018 (OriginLab Inc., Northampton, MA, USA) was applied to draw figures; Design Expert 13 (Stat-Ease, Inc., Minneapolis, MI, USA) software was used to study the results of the RSM optimization test.

## 4. Conclusions

RSM was employed to optimize the process conditions for ultrasonic-assisted extraction of SPP, including the ultrasonic time, temperature, power, and pH of the solution. The optimum process was obtained as follows: an ultrasonic time of 120 min, a power of 115 W, a temperature of 54 °C, pH of 10.5, and the extraction yield of SPP was 68.087%. Ultrasonic treatment could improve the functional characteristics of SPP, including the solubility, emulsifying activity and stability, WHC and OHC, foaming activity and stability, gel property, and flavor compounds adsorption capacity of SPP, which were closely related to the enhanced surface hydrophobicity induced by ultrasonic treatment. Although ultrasonic extraction optimization improved the extraction efficiency and functional characteristics of SPP, scaling up laboratory-level ultrasonic equipment to an industrial scale remains challenging. Issues such as uneven mass transfer and energy dissipation may compromise the stability and cost-effectiveness of large-scale production. Future researches should evaluate the feasibility of industrial-scale production of SPP and explore its applications in functional foods by investigating its nutritional profile and bioactive properties.

## Figures and Tables

**Table 1:** Design and results of the Box−Behnken experiment.

Serial Number	A	B	C	D	Extraction Yield%
1	−1	−1	0	0	60.39 ± 0.34
2	1	−1	0	0	63.18 ± 0.33
3	−1	1	0	0	64.67 ± 0.19
4	1	1	0	0	65.97 ± 0.01
5	0	0	−1	−1	64.12 ± 0.15
6	0	0	1	−1	59.4 ± 0.23
7	0	0	−1	1	63.14 ± 0.11
8	0	0	1	1	65.52 ± 0.46
9	−1	0	0	−1	58.73 ± 0.42
10	1	0	0	−1	66.74 ± 0.26
11	−1	0	0	1	64.96 ± 0.31
12	1	0	0	1	64.89 ± 0.45
13	0	−1	−1	0	64.85 ± 0.12
14	0	1	−1	0	64.62 ± 0.31
15	0	−1	1	0	59.45 ± 0.45
16	0	1	1	0	62.06 ± 0.11
17	−1	0	−1	0	64.58 ± 0.16
18	1	0	−1	0	65.97 ± 0.1
19	−1	0	1	0	61.55 ± 0.27
20	1	0	1	0	62.46 ± 0.16
21	0	−1	0	−1	55.78 ± 0.26
22	0	1	0	−1	64.31 ± 0.5
23	0	−1	0	1	66.57 ± 0.32
24	0	1	0	1	60.48 ± 0.32
25	0	0	0	0	68.81 ± 0.25
26	0	0	0	0	68.27 ± 0.04
27	0	0	0	0	68.03 ± 0.9
28	0	0	0	0	66.02 ± 0.22
29	0	0	0	0	67.14 ± 0.12

**Table 2:** Variance analysis of protein extraction yields from SPP.

Variance Source	Sum of Squares	Degree of Freedom	Mean Square	*p*-Value	Significance
model	255.73	14	18.27	<0.0001	**
A	17.11	1	17.11	0.0035	**
B	11.78	1	11.78	0.0115	*
C	23.63	1	23.63	0.0010	**
D	22.63	1	22.63	0.0012	**
AB	0.5550	1	0.5550	0.5382	
AC	0.0576	1	0.0576	0.8418	
AD	16.32	1	16.32	0.0041	**
BC	2.02	1	2.02	0.2490	
BD	53.44	1	53.44	<0.0001	**
CD	12.60	1	12.60	0.0094	**
A2	12.99	1	12.99	0.0086	**
B2	54.00	1	54.00	<0.0001	**
C2	31.73	1	31.73	0.0003	**
D2	43.73	1	43.73	<0.0001	**
Residual error	19.51	14	1.39		
Misfit term	14.72	10	1.47	0.4555	
Pure error	4.79	4	1.20		
synthesis	275.24	28			

Note: “**” indicates that the effect is extremely significant (*p* < 0.01), and “*” indicates that the effect is significant (*p* < 0.05).

**Table 3:** Effects of ultrasonic treatment on the physical and chemical properties of SPP.

SPP	Solubility/(g/L)	WHC/%	OHC/%	Foaming Capacity/%	Foam Stability/%	EAI/(m^2^/g)	ESI/%
Untreated	52.02 ± 0.46 ^b^	2.03 ± 0.02 ^b^	1.65 ± 0.03 ^b^	57.98 ± 2.32 ^b^	65.70 ± 0.37 ^b^	2.55 ± 0.90 ^b^	28.05 ± 0.77 ^b^
Ultrasonic treatment	65.15 ± 0.83 ^a^	2.21 ± 0.51 ^a^	1.93 ± 0.07 ^a^	113.33 ± 0.87 ^a^	78.41 ± 0.27 ^a^	4.70 ± 0.09 ^a^	50 ± 0.50 ^a^

Note: Data were expressed as means ± SEM. Labeled characters with different letters represent significant differences at *p* < 0.05.

**Table 4:** Effect of ultrasonic treatment on the secondary structure content of SPP.

Secondary Structure	Untreated (%)	Ultrasonic Treatment (%)
α-helix	0.40 ± 0.88 ^b^	6.00 ± 1.22 ^a^
β-sheet	77.10 ± 3.57 ^a^	63.60 ± 3.48 ^b^
β-turn	0.2 ± 0.21 ^b^	8.80 ± 1.50 ^a^
Random coil	22.30 ± 2.77 ^a^	21.60 ± 2.54 ^a^
Total	100%	100%

Note: Data were expressed as means ± SEM. Labeled characters with different letters represent significant differences at *p* < 0.05.

**Table 5:** Effect of ultrasonic treatment on texture properties of SPP gel.

SPP	Hardness/N	Elasticity/mm	WHC of the Gel/%
Untreated	16.87 ± 0.73 ^b^	1.26 ± 0.16 ^b^	37.21 ± 1.14 ^b^
Ultrasonic treatment	17.89 ± 1.45 ^a^	1.75 ± 0.32 ^a^	48.71 ± 1.20 ^a^

Data were expressed as means ± SEM. Labeled characters with different letters represent significant differences at *p* < 0.05.

**Table 6:** Factors and levels of the single-factor test.

Level	Ultrasonic Time/min	Ultrasonic Power/W	Solution pH	Ultrasonic Temperature/°C
−1	60	90	10	50
0	90	108	11	60
1	120	126	12	70

## Data Availability

The original contributions presented in this study are included in the article. Further inquiries can be directed to the corresponding author.
